# Anticancer Activity of Curcumin-Loaded PLGA Nanoparticles on PC3 Prostate Cancer Cells

**Published:** 2017

**Authors:** Seyed Saeed Azandeh, Mohammadreza Abbaspour, Ali Khodadadi, Layasadat Khorsandi, Mahmoud Orazizadeh, Abbas Heidari-Moghadam

**Affiliations:** a *Cell and Molecular Research Center, Faculty of Medicine, Ahvaz Jundishapur University of Medical Sciences, Ahvaz, Iran. *; b *Targeted Drug Delivery Research Center, School of Pharmacy, Mashhad University of Medical Sciences, Mashhad, Iran.*; c *Department of Immunology and Cancer, Petroleum Pollutants, Research Center, Ahvaz Jundishapur University of Medical Sciences, Ahvaz, Iran.*

**Keywords:** Programmed cell death, Prostate cancer, Curcumin, Nanoparticles, PLGA

## Abstract

Curcumin (Cur) has been found to be very efficacious against many different types of cancer cells. However, the major disadvantage associated with the use of Cur is its low systemic bioavailability. Our present work investigated the toxic effect of encapsulation of Cur in PLGA (poly lactic-coglycolic acid) nanospheres (NCur) on PC3 human cancer prostate cell. In the present study, we have investigated the effects of NCur on growth, autophagia, and apoptosis in PC3 cells, respectively, by MTT assay, fluorescence microscopy, and Flow cytometry. MTT assays revealed that the NCur at the concentration of 25 µg/mL for 48 h were able to exert a more pronounced effect on the PC3 cells as compared to free Cur. Apoptotic index was significantly increased in NCur-treated cells compared to free Cur. The percentage of autophagic cells (LC3-II positive cells) was also significantly increased in NCur treatment in comparison to free Cur. These data indicate that the NCur has considerable cytotoxic activity more than Cur on PC3 cell lines, which is mediated by induction of both apoptotic and autophagic processes. Thus, NCur has high potential as an adjuvant therapy for clinical application in prostate cancer.

## Introduction

Prostate cancer is the most frequently diagnosed cancer and the leading cause of cancer deaths among males worldwide. The best lines of defense, radiation therapy, and chemotherapy are unsatisfactory due to the untoward side effects on healthy cell and the problem of drug resistance (1-4). Searching for new compounds for the treatment of cancer is the aim of numerous studies, and many works are focused on plant-derived compounds that have curative potential. Curcumin (Cur) is a phenolic compound with yellow color from the plant, *Curcuma longa*, which has been used as a kind of traditional Asian medicine for centuries (1, 5).

Cur has many beneficial effects including anti-inflammatory, antioxidant, anticancer, antimicrobial, and anti-hyperlipidemic (6-10). Cur has also been shown to possess potent anti-neoplastic activity against a number of tumors including prostate, breast and colon cancer (11, 12). However, lack of aqueous solubility, rapid metabolism and conjugation in the liver has limited systemic bioavailability of Cur (13). Cur is lipophilic in nature. When nanoparticles of Cur is formulated with some amphiphilic polymer or phospholipids, Cur partitions and gets encapsulates into the hydrophobic core of nanoparticles which not only enhance its bioavailability but also increase its stability by protecting them from the influence of metabolizing enzymes (14). 

Polymers can be modified to have altered surface properties of the formulated nanoparticles. Different functional groups can be covalently or non-covalently conjugated with the polymeric chains to increase the mean residence time of the nanoparticles in the gastrointestinal mucosa (15). The advantages of such formulations are their low toxicity, high stability providing longer circulation and smaller size which attribute them increased cellular permeability for passive targeting of solid tumor tissue site with enhanced permeation and retention effect (16). It has been revealed that nanoparticle encapsulation improves oral bioavailability of Cur up to 9-folds compared to that of free Cur (17). Poly lactic-co-glycolic acid (PLGA) is extensively utilized for the development of polymeric nanoparticle of Cur (18). Xie *et al*. have shown that PLGA nanoparticles improve the oral bioavailability of Cur in rats (19). Yallapu *et al.* have shown that Cur loaded PLGA can inhibit proliferation of prostate cancer cells (18).

Defects in cancer cell death are the most frequent causes of therapeutic resistance, and thus exploring cancer cell death might inform development of strategies to overcome therapeutic resistance (20). In the present study, anticancer effects of Cur encapsulated in PLGA (NCur) on PC3 prostatic cancer cell line were investigated by assessment of cell viability, apoptosis and autophagy.

## Experimental

*Materials*

Poly (d,l-lactic-co-glycolic acid) (PLGA) with co-polymerization ratio 50:50 (lactic/glycolic), Polyvinyl alcohol (PVA) Mw ≈ 130,000, curcumin, 3-(4, 5dimethylthiazol-2-yl)-2, 5-diphenyltetrazolium bromide (MTT), dimethyl sulfoxide (DMSO), and DAPI (4′, 6-diamidino-2-phenylindole) were purchased from Sigma (Steinheim, Germany). Dulbecco’s Modified Eagel Medium (DMEM), Chloroform, ethanol, fetal bovine serum and Annexin V-FITC/propidium iodide assay kit were obtained from Gibco, Invitrogen (Carlsbad, CA, USA). Primary and secondary antibodies were purchased from Santa Cruz Biotechnology (Santa Cruz, CA, USA).

*Preparation of NCur*

Curcumin loaded PLGA nanoparticles were prepared by solvent (s/o/w) evaporation technique. Briefly, 60 mg of the PLGA were dissolved in 1 mL chloroform as an oil phase (organic solution). Free Cur (6 mg) was added to the PLGA/chloroform solution and sonicated to produce the s/o primary emulsion. To form the final s/o/w emulsion this emulsion was then added to a solution of 2% PVA and ethanol (1:1) and again was sonicated at 55 W for 2 min. To evaporation (remove the organic phase) of solvent (chloroform) s/o/w emulsion was sonicated and agitated by stirrer for 5-6 h. The sample was then centrifuged at 15000×g for 10 min and washed 2-3 times with distilled water. It was then freeze dried for 24 h to obtain the dry powder. The nanoparticles were stored at 4 °C for further use (16, 21).

*Characterization of NCur*

For characterization of NCur, encapsulation efficiency and particle size of the nanoparticles were determined. The encapsulation efficiency of the nanospheres was determined by analyzing the supernatant of the final emulsion once the nanospheres were removed from it by centrifugation at 15000×g for 15 min. For the estimation of Cur present in the supernatant, the absorbance was measured spectrophotometrically at 425 nm and the amount of drug present was calculated from calibration curves of concentration versus absorbance with known standards of the drug. Encapsulation efficiency (EE %) and Cur loading were calculated using below formula (16, 21).

$\mathrm{Encapsulation efficiency}\left(\%\right)=\frac{\mathrm{Amount of Cur in the nanoparticle}}{\mathrm{Initial amount of Cur}}\times 100$

$\mathrm{Cur loading}(\%)=\frac{\mathrm{Amount of Cur in the nanoparticle}}{\mathrm{Total number of nanoparticle}}\times 100$

Amount of Cur in the nanoparticles = Total amount of Cur- free Cur

Atomic force microscopy (AFM) method was used to determine the size and morphology of the synthesized NCur.

*Experimental design*

The human PC3 cell line was purchased from Pasteur Institute of Iran. PNT2 cell line (Normal human prostate epithelium) was purchased from sigma (Cat number: 95012613). Control group was untreated cells. Experimental groups were treated by 25 μg/mL of Cur and 25 μg/mL of NCur for 48 h, respectively. The dose of NCur was selected based on the results of previous studies (16) and our pilot study. Briefly, before the experiment, we examined different doses of NCur (1, 5, 25 and 50 µg/mL) for 24 and 48 h to determine the best one for anticancer action and 3 flasks were used for each dose. By using MTT assay, the percentage of viable cells was significantly decreased at the concentration of 25 and 50 µg/mL (results not shown). There was no significant difference between concentration of 25 and 50 µg/mL. Thus, we used Cur and NCur at the dose of 25 µg/mL in this study. Prior to beginning each test, manual cell counting by using a Hemacytometer was performed in order to standardize cell concentration between samples to minimize error and variation in downstream results (22).

*MTT assay*

MTT(3-(4,5-Dimethylthiazol-2-yl)-2, 5-diphenyltetrazolium-bromide) assays were used to compare the effect of NCur with Cur on cell viability and proliferation as previously described (23). Briefly, PC3 and PNT2 cells were seeded in 96-well plates (1 × 10^4^ cells/well). After treatment, the cells were maintained with culture media for 48 h. MTT (0.5 mg/mL) was then added to each well, and cells were further incubated for 4 h at 37 °C. Supernatants were then removed, and 700 µL of DMSO were added to each well to dissolve the formazan product. Absorbance at 540 nm was measured using a microplate reader (BioRad, Hercules, CA). Optical density values of the control cells were calculated as 100% viability. Because absorbance is in proportion to the number of living cells in a sample, the MTT assay reflects the extent of cell proliferation (24**)**.

*DAPI staining*

The PC3 and PNT2 cells (10^4^ cells/mL) were grown on cover slips after treatment with NCur or Cur for 48 h. Nuclei changes of apoptosis were assayed by DAPI (4,6-diamidino-2-phenylindole) staining as previously described (25). Following washed with PBS, the cells were fixed with cold methanol/acetone for 5 min at room temperature, washed with PBS, and then incubated with DAPI solution (2 mg/mL in PBS) for 10 min at room temperature. Fluorescence images were captured using a fluorescence microscope (Olympus, Japan).

*Immunocytochemistry*

The PC3 and PNT2 cells (10^4^ cells/ mL) after treatment deposited on a glass cover slip were fixed for 15 min in 4% paraformaldehyde in PBS at 4 °C. Nonspecific binding was blocked with 1% bovine serum albumin-PBS for 15 min. Anti-LC3-II antibody (sc-16755, Santa Cruz) at 1/100 dilution was applied overnight at 4 °C. After two washes with PBS, the sections were incubated with FITC-conjugated anti-mouse secondary antibody (sc-2356, Santa Cruz) for 1 h at room temperature. After washing, the cells were incubated with DAPI at dilution 1: 1000 in PBS for 10 min. Fluorescence images were captured using a fluorescence microscope (Olympus, Japan). The percentage of LC3-II positive cells was calculated by dividing the number of LC3-II positive cells in a randomly microscopy field by the total number of cells in that field, and the result was multiplied by 100. There were at least 3 slides for different groups. Ten randomly field were evaluated for each slide (26).

**Figure 1:**
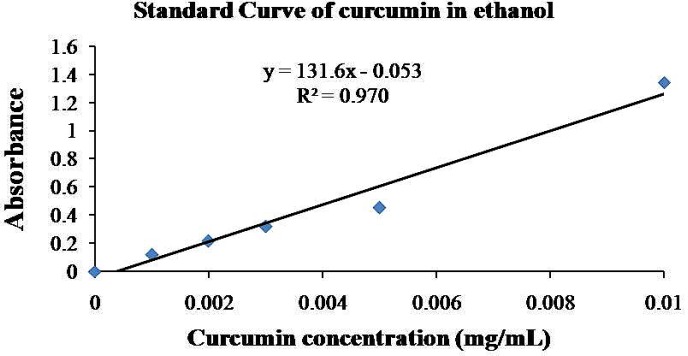
Standard curve of Cur-loaaded PLGA nanosphers. The loading was found to be 97%.

**Figure 2:**
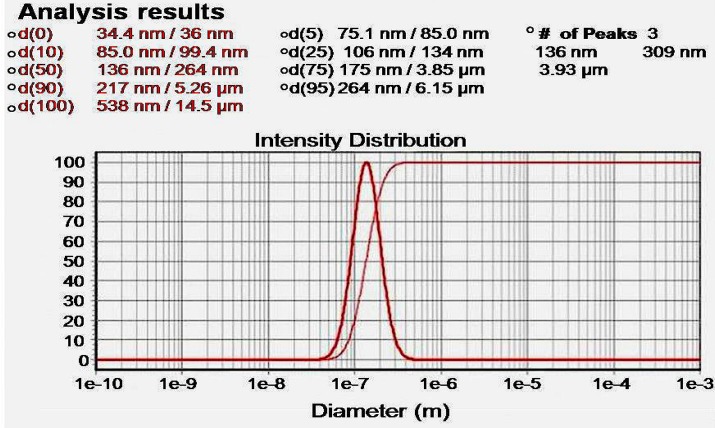
Particle size distribution of Cur-loaaded PLGA nanosphers. The mean particle diameter was found to be 136 nm.

**Figure 3:**
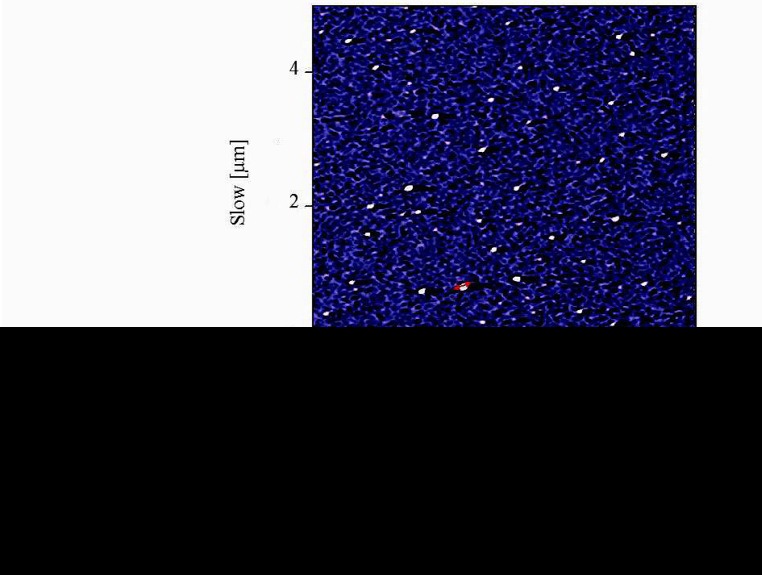
AFM image of nanoparticles showed distinct spherical particles in size range between 100 and 200 nm.

**Figure 4:**
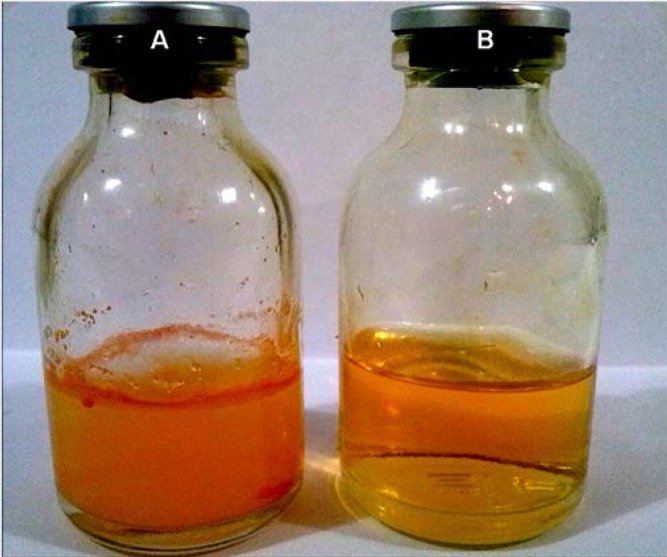
Free Cur in water (A) and Cur-loaded PLGA nanosphers in water (B).

**Figure 5:**
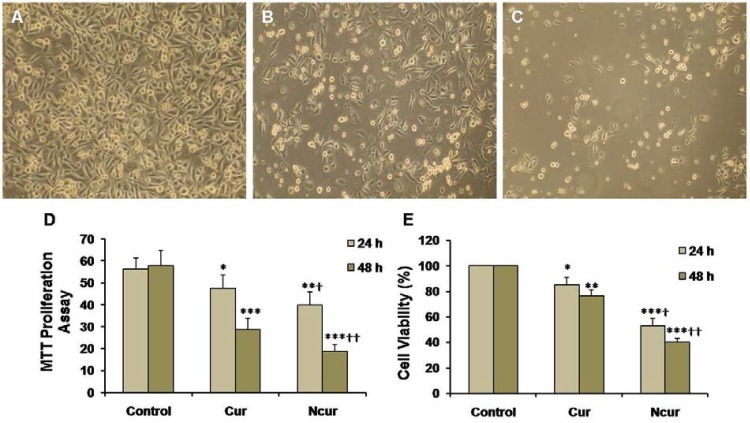
PC3 cell cultures of control (A), Cur (B), and NCur (C) groups (magnifications: × 100). MTT proliferation assay (D) and percentage of cell viability (E) in control and experimental groups are shown. All assays were performed in triplicate, and the mean ± standard deviations are shown. *p < 0.05, **p < 0.01, ***p < 0.001,^ †^p < 0.01, ^††^p < 0.001; * and † symbols respectively indicate comparison to control and Cur groups

**Figure 6:**
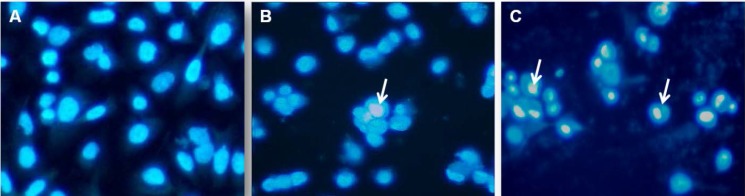
DAPI staining of control and experimental groups. (A) Control group, (B) Cur group, (C) NCur group. Arrows indicate chromatin condensation. Magnifications: × 400

**Figure 7.**
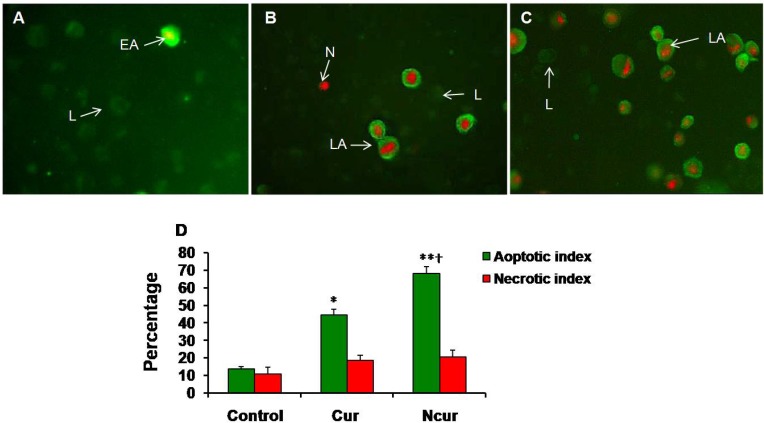
Immunoflorecent microscopy of Annexin/PI staining in control and experimental groups (Magnifications: × 250). (A) Control group; (B) Cur group; (C) NCur group. EA: early apoptosis (Cell membrane is strongly stained with FITC), LA: late apoptosis (Cell membrane is strongly stained with FITC and Nucleus is stained by PI), N: Necrosis (Nucleus have red stain), L: Live (Cells have slightly green stain). (D) Apoptotic and necrotic indexes of control and experimental groups. Values are expressed as mean ± SD. *p < 0.01, **p < 0.001,^ †^p < 0.01; * and † symbols respectively indicate comparison to control and Cur groups

**Figure 8:**
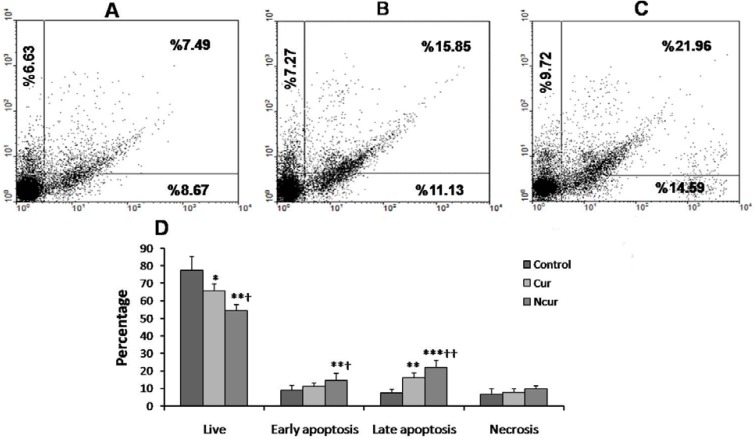
Flow cytometry of Annexin/PI staining in control and experimental groups. (A) control group, (B) Cur group, (C) NCur group. The lower left quadrant (Annexin V-FITC-/PI−) was considered as live cells. The lower right quadrant (Annexin V-FITC+/PI−) was considered as early-stage apoptotic cells, the upper right quadrant (Annexin V-FITC+/PI+) was considered late-stage apoptotic cells, and the upper left quadrant (Annexin V-FITC−/PI+) was considered as necrotic cells. (D) Summaries of changes in the percentage of viable cells, necrotic and apoptotic cells. All assays were performed in triplicate, and the mean ± standard deviations are shown. *p < 0.05, **p < 0.01, ***p < 0.001,^ †^p < 0.01, ^††^p < 0.001; * and † symbols respectively indicate comparison to control and Cur groups

**Figure 9:**
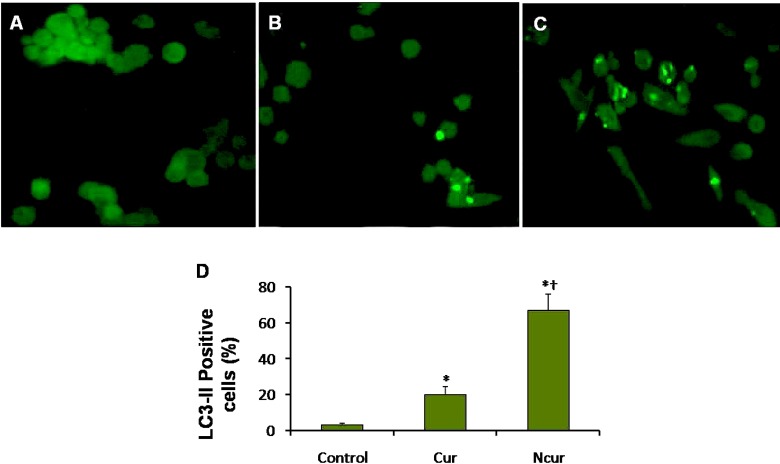
Immunoflorecent microscopy of PC3 cells staining in control and experimental groups. Autophagosomes have light green staining. (A) Control group, (B) Cur group, (C) NCur group. Magnifications: ×400. (D) percentage of LC3-II positive cells. All assays were performed in triplicate, and the mean ± standard deviations are shown. *p < 0.001, ^†^p < 0.001; * and † symbols respectively indicate comparison to control and Cur groups

**Table 1:** MTT assay of PNT2 cells in various groups. Percentage of cell viability in 24 and 48 h are presented

**Groups **	**24 h **	**48 h **
Control	100 ± 0.01	100 ± 0.02
Cur	98.1 ± 0.35	100.3 ± 1.32
NCur	99.4 ± 0.35	101.2 ± 1.65

*Annexin V-FITC/propidium iodide apoptosis assay*

PC3 and PNT2 cells were placed in a six-well culture plate and treated with Cur or NCur for 48 h as experimental groups. Control group was untreated cells. Normal, apoptotic and necrotic cells were distinguished using an Annexin V-FITC/propidium iodide assay kit according to the manufacturer’s instructions. Briefly, after the incubation period, the cells (1.0 × 10^6^) were washed in cold PBS. The washed cells were re-centrifuged, discarded the supernatant, and resuspended in 1X annexin-binding buffer. Twenty five μL of the Annexin V conjugate and 2 μL of the 100 μg/mL propidium iodide (PI) working solution were added to each 100 μL of cell suspension. The cells were incubated at room temperature for 15 min and then were washed with 1X Annexin-Binding buffer and deposited onto slides. 

The fluorescence was observed by using appropriate filters. The cells were separated into three groups: live, apoptotic (early and late), and necrotic cells. Live cells showed only weak Annexin V staining of the cellular membrane. Early apoptotic cells showed a significantly higher degree of surface labeling. Late apoptotic cells showed both membrane staining by Annexin V and strong nuclear staining from the PI. Necrotic cells showed only nuclear staining from the PI. Apoptotic index and necrotic index were calculated by dividing the number of apoptotic or necrotic cells in a randomly microscopy field by the total number of cells in that field, and the result was multiplied by 100. The Apoptotic indexes and necrotic indexes of 10 randomly field were evaluated and the mean apoptotic index and necroptotic index of each case were calculated (24). Flow cytometry by using this kit was also performed. Briefly, the cells were exposed to Cur or NCur in 6-well plates for 48 h. After exposure, cells were trypsinized and centrifuged at 1000 rpm, the cell pellet was washed with PBS once and re-suspended in 100 mL of binding buffer, then incubated with 2 mL Annexin V-FITC for 10 min, which was followed by staining with 2 mL PI. Then, the samples were diluted with 400 mL binding buffer and analyzed with a Flow cytometer (Becton Dickinson, San Jose, CA), and at least 10000 cells were counted for each sample. The cell population of interest was gated on the basis of the forward and side-scatter properties. The different labeling patterns in the Annexin V/PI analysis identified the different cell populations where FITC negative and PI negative were designated as viable cells; FITC positive and PI negative as early apoptotic cells; FITC positive and PI positive as late apoptotic cells and FITC negative and PI positive as necrotic cells. The data analysis was performed using WinMDI 2.9 software.

*Data Analysis*

Comparisons of multiple (> 3) group means were performed using one-way analyses of variance and post-hoc procedures based on Newman-Keuls tests. Student’s t tests were used for comparisons of two group means. A p-value less than 0.05 is considered statistically significant.

## Results

*Characterization of NCur*

The particle size distribution showed a range of 100 nm to 200 nm, with the mean particle size being 136 nm (Figure 1). The encapsulation efficiency of Cur-loaded PLGA nanospheres was 97 ± 0.45%. Standard curve is shown in Figure 2. AFM revealed the size and morphology of the synthesized NCur. The complexes appear spherical with a mean size between 100 and 200 nm as can be seen in Figure 3. In addition, the NCur-loaded PLGA nanospheres prepared were completely dispersed in aqueous media with no aggregates as opposed to free Cur which exhibits poor aqueous solubility (Figure 4).

*Cell viability and proliferation*

MTT assays showed that exposure of PC3 to Cur induced a significant decrease in cell viability (p < 0.01). Cell viability in the NCur treated cells were significantly decreased compared to Cur treatment (p < 0.05). Viability of PNT2 cells were not changed in response to Cur or NCur (Table 1). MTT proliferation assay also showed that NCur significantly inhibited growth of PC3 cells in comparison of Cur. These results are shown in Figure 5.

*DAPI staining*

In order to further elucidate NCur cell death in PC3 cells, we analyzed cell nuclear morphology with DAPI staining after 48 h of Cur or NCur treatment. As shown in Figure 6 NCur-treated PC3 cells exhibited chromatin condensation which indicated cell apoptosis. Apoptotic index in PNT2 cells was not significantly changed in response to Cur or Ncur (the results not shown).

*Annexin V-FITC/propidium iodide apoptosis assay*

A few necrotic and apoptotic cells were observed in control group by using a fluorescence microscope. In Cur-treated cells, a large number of PC3 cells showed apoptosis. In NCur- treated cells, the percentage of apoptotic cells (apoptotic index) was significantly increased in comparison to free Cur group (p < 0.01). There were not significant changes in necrotic index between control and experimental groups. Similar results were obtained by using Flow cytometry method. These results are shown in Figures 7 and 8. Apoptotic and necrotic index in PNT2 cells were not significantly changed in response to Cur or Ncur (the results not shown).

*Autophagy*

Autophagy was assessed by using immunocytochemistry for LC3-II and AO staining for detect autophagosomes. Autophagosomes (LC3 positive vesicles) showed light green appearance. A few LC3 positive cells were observed in control group. The percentage of LC3-II positive cells in Cur treatment was significantly increased. The percentage of LC3-II positive cells in NCur treatment was significantly increased in compared to Cur treated cells. These results are shown in Figure 9. In PNT2 cells, the percentage of LC3-II positive cells was not significantly changed in response to Cur or Ncur (the results not shown).

## Discussion

This study demonstrated that encapsulation of Cur in PLGA could effectively enhance its anticancer effects. NCur could effectively inhibit the growth of PC3 cells and induced apoptosis and autophagy in these cells. NCur could reduce viability of PC3 cells. Nair *et al.* showed that cellular uptake of Cur encapsulated in PLGA in human epithelial cervical cancer cells (HeLa) were enhanced compared to free Cur. They have also proved that NCur have more pronounced antitumor activity by using anti-proliferative studies (MTT assay) and Annexin V/propidium iodide staining (16). Mukerjee *et al.* by using cell viability studies have demonstrated that Cur encapsulated in PLGA is able to exert a more pronounced effect on the prostate cancer cells as compared to free Cur (21).

Other studies of Cur formulations such as micellar aggregates of cross-linked and random copolymers of N-isopropylacrylamide, with N-vinyl-2- pyrrolidone and poly (ethyleneglycol)

monoacrylate and self-assembling methoxy poly(ethylene glycol)–palmitate Cur nanocarrier have shown to exhibit similar growth inhibition to that of free Cur (27-29).

A cationic poly (vinyl pyrrolidone) -Cur conjugate has been judged by MTT assay to be more potent in L929 fibroblast cells over free Cur (30). Tang *et al.* have demonstrated that polycatocol-Cur conjugate is highly cytotoxic to ovarian cancers (SKOV-3 and OVCAR-3) and MCF-7 breast cancer cell lines (31). 

As shown in results, NCur can effectively increase apoptosis in PC3 cells. Most current anticancer drugs kill actively dividing cells by the induction of apoptosis (32). Apoptotic cell death involves a series of events leading to characteristic changes in cell morphology, including loss of cell membrane asymmetry, nuclear fragmentation, chromatin condensation, chromosomal DNA fragmentation, and activation of caspases (33). In DAPI staining we observed that NCur considerably induced chromatin condensation and nuclear fragmentation. The results of annexin V/PI assay revealed that apoptosis, not necrosis, was the predominant mechanism in NCur**-**induced cytotoxicity. 

Unfortunately, cancer cells often acquire resistance to agents that activate the apoptotic pathway (36). Thus activation of other death pathways may be helpful to management of cancer therapy. Autophagy has recently gained much attention for its paradoxical roles in cell survival and cell death, particularly in the pathogenesis as well as the treatment of cancer (34, 35). Whether autophagy enables cells to survive or enhances their death is context-driven, depending on the type of stimuli, nutrient availability, organism development, and apoptotic status (36). 

Autophagy induced during starvation, growth factor deprivation, hypoxia, endoplasmic reticulum stress, and microbial infection can prevent cell death (37). However, it can be also associated with cell death due to excessive mitophagy, leading to loss of mitochondrial membrane potential (Δψ_m_), caspase activation, and lysosomal membrane permeabilization (38). 

As shown in results, NCur can effectively increase percentage of LC3-II positive PC3 cells. During autophagosome formation, cytosolic microtubule-associated protein light chain 3-I (LC3-I) is conjugated with phosphatidylethanolamine and converted to LC3-II. This phosphatidylethanolamine-conjugated LC3-II, detectable by immunoblotting, is present specifically on isolation membranes and autophagosomes and therefore serves a second and widely accepted approach to monitoring autophagia (39). 

During autophagy, parts of the cytoplasm are digested by lysosomes, thereby providing metabolites that are used for cell homeostasis. Although autophagy is a process with a major role in cell survival, it is also capable of inducing cell death characterized by extensive digestion of intracellular organelles (40). Importantly, a number of small molecules (including several anticancer drugs) activate autophagy both *in-vivo* and *in-vitro* in cancer cells, indicating the importance of this biological process in the development of chemotherapeutics (41, 42). The importance of autophagic cell death induced by some anticancer therapeutic agents in relation to the regression of cancer cell growth through multiple mechanisms is recently emerging. For example the cytotoxic compound docosahexaenoic acid can induce both apoptosis and autophagic cell death (43). 

The exact mechanism of NCur effects on PC3 cells is not obtained from this study. Our results have demonstrated that NCur enhances both apoptosis and autophagia processes in PC3 cells. Additionally, viability of PC3 cells was considerably reduced by NCur. These findings indicate that NCur can induce both type I and II programmed cell death. 

Mouratidis *et al.* showed that Lupulone, a β-acid derived from hop extracts, induced apoptosis and autophagia in PC3 and DU145 prostate cancer cells (44). Ullén *et al.* have also demonstrated that sorafenib reduces cell viability and induces apoptosis and cellular autophagy (45). In cancer, an autophagy paradox has emerged in which survival and death are context specific, particularly due to complex interactions between autophagic and apoptotic pathways. Accordingly, cancer therapies have been reported to have opposing effects on cell death. Photodynamic therapy promotes autophagic cell death in apoptosis-deficient cancer cells (46), whereas sulforaphane-induced autophagy in PC3 and LNCaP cells is protective (47). Furthermore, manipulation of autophagy can sensitize tumor cells to subsequent treatments. Induction of autophagy by an mTOR inhibitor increased prostate cancer cell susceptibility to irradiation (48). 

Additionally, excessive autophagy will also inevitably trigger autophagic cell death or type II programmed cell death. Due to emerging links between autophagic aberrations and cancer, there is rapidly growing support that this type II programmed cell death may also function as a suppressor of tumorigenesis (40).

## Conclusion

In summary, this study has demonstrated that NCur stimulates the both type I and II programmed cell death and appear to be good candidates for further preclinical studies of prostate cancer treatment. Extrapolation of these data to the human situation is not appropriate. However, this information does provide a stimulus for true clinical investigations.
